# Real-World Clinical Outcomes and Adverse Events in Patients with Chronic Lymphocytic Leukemia Treated with Ibrutinib: A Single-Center Retrospective Study

**DOI:** 10.3390/medicina59020324

**Published:** 2023-02-09

**Authors:** Ana-Maria Moldovianu, Razvan Stoia, Mariana Vasilica, Iulia Ursuleac, Sorina Nicoleta Badelita, Andra Alina Tomescu, Oana Diana Preda, Alexandru Bardas, Mihaela Cirstea, Daniel Coriu

**Affiliations:** 1 Department of Hematology and Bone Marrow Transplant, Fundeni Clinical Institute, 022328 Bucharest, Romania; 2 Department of Hematology, University of Medicine and Pharmacy “Carol Davila”, 050474 Bucharest, Romania

**Keywords:** chronic lymphocytic leukemia (CLL), ibrutinib, real-world, Bruton tyrosine kinase inhibitor, adverse events

## Abstract

*Background and Objectives*: The treatment of chronic lymphocytic leukemia (CLL) has acquired new targeted therapies. In clinical trials, ibrutinib improved outcomes safely. Real-world data called for a reappraisal of ibrutinib strategies. We report on a single center’s experience with ibrutinib monotherapy, aiming to explore the outcomes, tolerability, and prognosis of CLL patients in routine clinical practice. *Materials and Methods*: Data were collected from all CLL patients treated with ibrutinib at Fundeni Clinical Institute, Bucharest, Romania, between January 2016 and June 2021. *Results*: A total of one hundred twenty-three CLL adult patients were treated with ibrutinib. Of the patients, 87% had relapsed/refractory CLL. The median age at ibrutinib initiation was 65 years; 44.7% of patients were staged Rai III/IV. At 32-month median follow-up, the median progression-free survival (PFS) was 50 months, the overall survival (OS) was not reached, and the overall response rate (ORR) was 86.2%. The age or number of previous therapies did not impact outcomes or tolerability. An Eastern Cooperative Oncology Group performance status (ECOG PS) score ≥ 2 and shorter time from initiation of last therapy (TILT) before ibrutinib predicted inferior PFS. Baseline characteristics had no impact on the OS except for TILT in R/R CLL patients. Drug-related adverse events (AEs) of any grade and grade ≥ 3 AEs were reported in 82.1% and 30.9% of the patients, respectively. Infections were the most common AEs (29.3%). Drug discontinuation was permanent in 43.9% of patients, mainly due to disease progression (17.1%) and toxicity (8.9%). Patients with a Cumulative Illness Rating Scale (CIRS) score ≥ 6 had a higher risk for toxicity-related discontinuation. An ECOG PS ≥ 2 predicted an increased rate of permanent discontinuation and grade ≥ 3 AEs. *Conclusions*: The outcomes of this study align with the results from ibrutinib clinical trials. Our study demonstrated that poor patient fitness, early relapse before ibrutinib, and permanent ibrutinib discontinuation are essential outcome determinants. Patient comorbidity burden and fitness were significant predictors for ibrutinib intolerance.

## 1. Introduction

Traditionally, systemic chemoimmunotherapy was the mainstay in treating chronic lymphocytic leukemia (CLL). Strategies with fludarabine, cyclophosphamide, and rituximab (FCR), or bendamustine and rituximab (BR), may achieve sustained remissions [1,2]. However, toxicity and early relapse (i.e., within three years) [3,4] made compelling arguments for safer alternatives such as the novel targeted drugs.

Ibrutinib emerged as the first small-molecule inhibitor to selectively block the activity of the Bruton tyrosine kinase (BTK) [5,6,7], with excellent efficacy in CLL proved by no less than five clinical trials. Initially approved only in patients with relapsed/refractory (R/R) and deletion 17p (del 17p) CLL, ibrutinib was later endorsed as first-line therapy for all CLL patients [8,9] and soon followed by other oral mechanism-driven drugs (e.g., idelalisib, venetoclax, acalabrutinib). Currently, the more versatile and efficacious novel therapeutic agents have largely supplanted chemoimmunotherapy.

Randomized controlled trials (RCTs) and real-world studies provide distinct insight, sometimes contradictory, into ibrutinib therapy. Far from being mutually exclusive, they are complementary and should be used in tandem to secure optimum management [10]. Compared to RCTs evidence, several real-world studies found lower survival and higher discontinuation rates with ibrutinib [11,12,13,14,15,16], while others reported similar results [17,18,19,20]. Furthermore, the real-life use of ibrutinib has sparked controversy on several other counts, including treatment adherence, adverse events (AEs), long-term outcomes, and high-risk factors [13,14,15,16,20,21,22].

This real-world analysis aims to evaluate the clinical outcomes, the incidence of AEs, discontinuation rate, prognostic factors, and disease characteristics of CLL patients receiving ibrutinib as a single-agent therapy.

## 2. Materials and Methods

### 2.1. Study Oversight and Eligibility

We carried out a single-center observational retrospective study of one hundred twenty-three adult patients diagnosed with CLL and small lymphocytic lymphoma (SLL) who received ibrutinib from January 2016 until June 2021. After approval by the Institutional Review Board for Biomedical Research of Fundeni Clinical Institute, Bucharest, Romania (748/08.01.18), we identified patients through hospital medical records and files. Patients enrolled in clinical trials or with missing information related to the date of demise or the last follow-up were excluded.

### 2.2. Data Collection

All medical files containing day-to-day documentation were reviewed. Collected data included the time of CLL diagnosis, patient demographics, performance status (PS) assessed with Eastern Cooperative Oncology Group (ECOG) score, comorbidities, Rai and Binet stage, biological, genetic, and molecular characteristics, treatment regimens (i.e., before and after ibrutinib), AEs, dose adjustments, reasons for discontinuation, and survival.

The cumulative illness rating scale (CIRS) [23] score was computed to assess the burden of comorbidity. Follow-up continued until demise or data cut-off, whichever came first. Data on treatment effectiveness included duration of therapy and reasons for permanent cease. The best response was graded according to the International Workshop on CLL (iwCLL) 2018 [24]. Overall response rate (ORR) included partial remission (PR) and complete remission (CR) rates, defined by clinical and hematological criteria. In general practice, bone marrow biopsy and CT scan are not mandatory [24].

AEs were filed according to type, incidence, severity, and the need to reduce, temporarily, or permanently discontinue ibrutinib therapy. The Common Terminology Criteria for Adverse Events (CTCAE) version 5.0 were used to grade all AEs, apart from hematologic toxicity, which was classified with the iwCLL 2018 scale [24]. Temporary discontinuation was defined as a minimum period of 14 days without ibrutinib. We measured progression-free survival (PFS) from the start of treatment until disease progression or death and overall survival (OS) from the start of treatment until death from any cause.

### 2.3. Study Endpoints

The primary study endpoints were PFS and OS. Secondary endpoints included ORR and ibrutinib-related toxicity. We also evaluated the factors that affect survival and tolerability to ibrutinib therapy.

### 2.4. Statistical Analysis

Demographics and disease characteristics were summarized descriptively, focusing on treatment-naïve (TN), R/R CLL, and overall population. Quantitative variables were reported as the median and 25–75th interquartile range (IQR), while counts and percentages were used to describe qualitative variables. Comparison between categorical variables was performed using λ2 or Fischer’s exact test. Variables with statistical significance *p* < 0.2 in the univariate analysis were included in a multivariate model using the enter method. Multivariate logistic regression was performed to assess the factors associated with treatment discontinuation, dose modifications, temporary interruptions, and AEs. The Kaplan–Meier method and the log-rank test were used to estimate the duration of ibrutinib treatment, PFS, and OS. The multivariate cox proportional hazard regression was performed to assess the association between demographic (i.e., age), clinical (i.e., ECOG PS, CIRS, disease stage), and biological (i.e., genetic and molecular alterations) factors with PFS and OS. For all comparisons, a two-tailed *p* < 0.05 was considered significant. All statistical analyses were performed using IBM SPSS Statistics 20.0 for Windows (IBM Corp., Armonk, NY, USA).

## 3. Results

### 3.1. Study Population

Between January 2016 and June 2021, one hundred twenty-three adult patients diagnosed with CLL/SLL received ibrutinib treatment. Patient characteristics at the time of ibrutinib initiation are presented in Table 1. A total of sixteen (13%) patients received ibrutinib for frontline treatment and 107 (87%) for R/R disease. The overall median (IQR) age at the index date was 65 (58–71) years. Most patients had a CIRS score of fewer than 6 points (89.4%). CLL staging showed that 47.7% and 36.6% of patients presented Rai stage III/IV and Binet C disease. The prognostic molecular profile, including IGHV mutational status, del 17p, and TP53 anomalies, was available for 35.8%, 42.3%, and 36.6% of patients, respectively. Only 34.1%, 10.6%, and 12.2% of patients tested positive for unmutated IGHV, del 17p, and TP53 mutations, respectively.

### 3.2. Pre-Ibrutinib Treatment Regimens

Table 2 presents therapies administered before ibrutinib. Half of the patients received two or more therapy lines. A total of forty-two (39.3%) patients were administered fludarabine in the first and later lines of treatment. A total of Ninety-three (86.9%) patients received an anti-CD20 monoclonal antibody (anti-CD20) (i.e., rituximab, ofatumumab, obinutuzumab). The proportion of patients receiving both purine analog and anti-CD20 therapy was 37.4%.

### 3.3. Outcomes

The median (IQR) follow-up from the beginning of treatment was 32 (22–51) months for the whole cohort, 37 (24–53) months for the R/R CLL patients, and 19 (16–26) months for the TN patients. Based on the clinical evaluation of the patient best response, the ORR (CR and PR) was 87.5% when ibrutinib was used as frontline and 86% when it was used as a second or later line. Assessment of response according to the treatment line is summarized in Table 3.

At median follow-up, the median PFS for the R/R CLL patients was 50 months [95% confidence interval (CI): 42.3–57.7], while the median OS was not reached (NR) [95% CI: NR-NR]. The 24-month PFS and OS rates were 77.7% [95% CI: 69.5–85.9%] and 87.9% [95% CI: 81.5–94.4%], respectively (Table 4).

When ibrutinib was used in frontline therapy, the median PFS and OS were NR [95% CI: NR-NR]. The 24-month PFS and OS rates were 75.8% [95% CI: 50.9–100%] and NR [95% CI: NR-NR], respectively (Table 4).

The number of therapy lines received before ibrutinib initiation did not alter PFS and OS (Figure S1(A1,B1)). Similarly, the age at the time of ibrutinib initiation did not significantly influence the PFS (*p* = 0.6130) and OS (*p* = 0.489) (Figure S1(A2,B2)).

**Figure 1:**
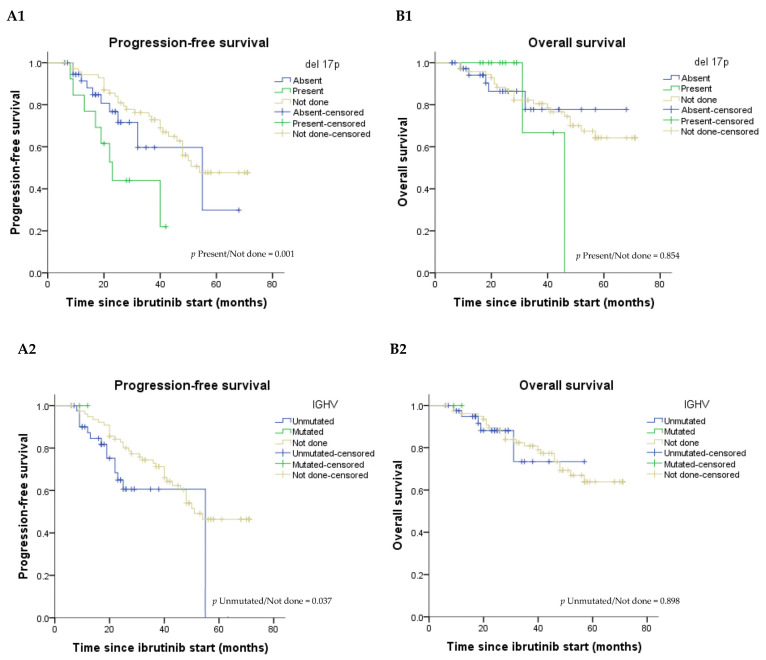
Kaplan–Meier estimated progression-free survival and overall survival curves according to the del 17p ((**A1**) and (**B1**), respectively) and IGHV status ((**A2**) and (**B2**), respectively). Abbreviations: del 17p, deletion 17p; IGHV, immunoglobulin heavy chain variable region genes.

Subgroup analysis revealed no difference in the PFS (*p* = 0.073) and OS (*p* = 0.888) between the del 17p positive and negative patients (Figure 1(A1,B1)). Compared to the patients with an unknown FISH test, del 17p patients had a hazard ratio (HR) for PFS of 3.26 [95% CI: 1.0–10.9] (*p* = 0.001) (Figure 1(A1)).

The log-rank tests showed that PFS and OS were not different in patients with other high-risk features, such as the unmutated IGHV (Figure 1(A2,B2) and TP53 mutations (Figure S2A,B)). After the inclusion of patients with unavailable molecular data, the PFS became significantly different between those with unmutated IGHV and those with unknown status (*p* = 0.037) (Figure 1(A2)).

**Figure 2:**
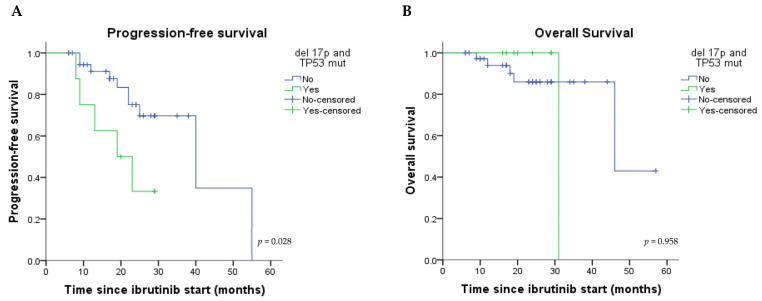
Kaplan–Meier estimated progression-free (**A**) and overall survival (**B**) curves according to the presence/absence of combined del 17p and TP53 mutations. Abbreviations: del 17p, deletion 17p; TP53 mut, TP53 mutations.

On the other hand, the combination of del 17p and TP53 mutations adversely impacted the PFS but not OS, with a 3.24 [95% CI: 1.06–9.94] higher risk for disease progression or death (*p* = 0.028) (Figure 2A,B).

**Figure 3:**
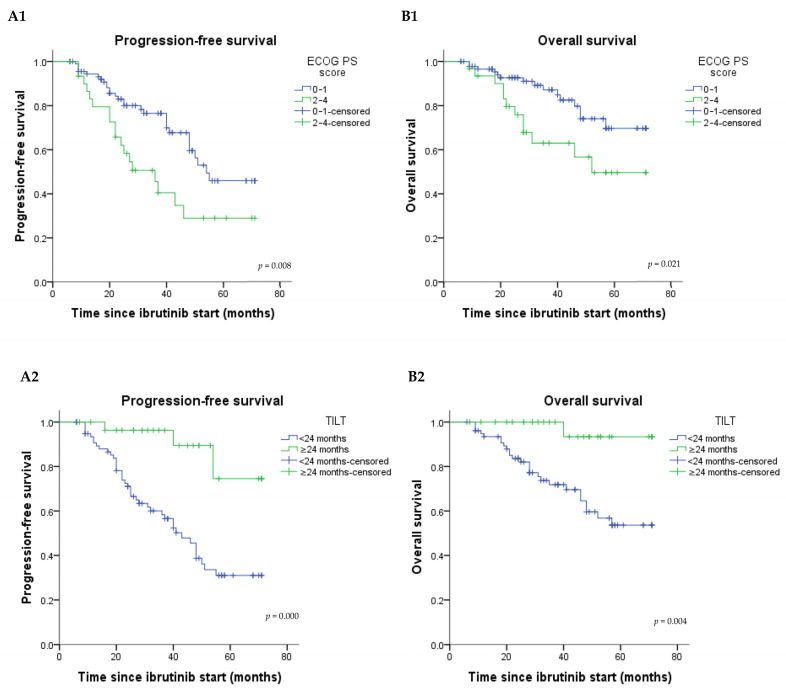
Kaplan–Meier plot showing the progression-free survival and overall survival curves according to the ECOG performance status (PS) score ((**A1**) and (**B1**), respectively) and time from initiation of the last treatment (TILT) before ibrutinib ((**A2**) and (**B2**), respectively). Abbreviations: ECOG, Eastern Cooperative Oncology Group; TILT, time from initiation of the last treatment (before ibrutinib).

In univariate analysis, patient gender, baseline cardiovascular comorbidities (i.e., hypertension, atrial fibrillation, cardiovascular disease), the Rai and Binet stage, beta2-microglobulin (B2M), and lactate dehydrogenase (LDH) did not alter the PFS or OS (data not shown). Conversely, an ECOG PS score from 2 to 4 significantly decreased PFS and OS (Figure 3(A1,B1)). Similar results were recorded with a time from initiation of the last therapy (TILT) before ibrutinib of fewer than 24 months (Figure 3(A2,B2)). The HR for progression or death in the group with ECOG score ≥ 2 was 2.18 [95% CI: 1.21–3.93] (*p* = 0.008) and increased to 6.17 [96% CI: 1.91–19.95] (*p* = 0.000) secondary to a response duration prior to ibrutinib therapy of fewer than 24 months. Comorbidities evaluated by CIRS at the start of ibrutinib did not significantly alter the outcomes, although a divergence in the PFS and OS curves was noted after 50 months of ibrutinib therapy (Figure S3A,B).

Table 5 describes the Cox proportional hazard regression model for baseline factors contributing to PFS and OS in an unadjusted and adjusted-for-other covariates version. Multivariate analysis showed that none of the covariates except for TILT could predict inferior OS in the R/R CLL patients. Contrarily, the ECOG status and TILT maintained their statistical significance as independent risk factors for PFS. We excluded the TN patients from this analysis, as they represent distinctive clinical and biological characteristics of the disease.

At the end of the follow-up, 27 patients died: 5 of disease progression and drug-related toxicity (i.e., infection), 3 of second primary malignancy (SPM), 1 secondary to Richter transformation (RT), 7 due to COVID-19 infection, and 6 due to an unrelated/unknown cause.

### 3.4. Ibrutinib Therapy

The median (IQR) time from diagnosis to ibrutinib therapy was 56 (30–80) and 6.5 (1.25–21.5) months for R/R and TN patients, respectively. Of the patients, 72.9% progressed or relapsed after a TILT of fewer than 24 months.

Ibrutinib was administered for a median (IQR) time of 32 (20–48), 19 (10–25), and 29 (18–45) months in the R/R, TN, and to the entire study population.

At median follow-up, the median Kaplan–Meier estimated ibrutinib treatment duration was 51 [95% CI: 36.46–65.54] months for R/R patients and NR [95% CI: NR-NR] in the TN group (*p* = 0.386).

At data cut-off, 54 (43.9%) patients permanently discontinued ibrutinib after a median (IQR) time of 22.5 (12–41) months. Table 6 presents the reasons for the cessation of ibrutinib therapy. The median (IQR) time to ibrutinib discontinuation due to AEs and CLL progression was 21 (6–37) and 23 (19–43) months, respectively.

A significant difference in median OS estimates (46 months vs. NR, *p* = 0.000) was observed between patients who discontinued and those who continued ibrutinib. Among 21 (17.1%) patients who discontinued ibrutinib due to disease progression, 13 received salvage therapy with venetoclax.

Subgroup analysis found no difference in OS between patients who stopped ibrutinib due to drug-emergent AEs versus other causes (*p* = 0.880), including disease progression (*p* = 0.37) (Figure S4A,B).

The causes of permanent discontinuation included atrial fibrillation (AF) (*n* = 3), infections (*n* = 2), bleeding (*n* = 2), viral B hepatitis (HBV) reactivation (*n* = 2), and ulcerative stomatitis (*n* = 2). A multivariate logistic regression test found an ECOG PS ≥ 2 and a CIRS score ≥ 6 independently associated with an increased risk for permanent discontinuation (Table 7 and Table S2).

Dose reduction was required in 35 (28.5%) patients. AEs were the main reason (90.9%), with neutropenia (*n* = 7), thrombocytopenia (*n* = 6), AF (*n* = 4), bleeding (*n* = 4), and maculopapular rash (*n* = 4) the most frequently in that order.

Ibrutinib was temporarily interrupted (i.e., at least 14 consecutive days) in 47 (38.2%) patients, with 36 (78.3%) necessitating only a one-time interruption. Among the patients who temporarily discontinued ibrutinib, 93.6% of cases were due to AEs, including infections (*n* = 11), grade ≥ 3 neutropenia (*n* = 8), bleeding (*n* = 7), HBV reactivation (*n* = 5), and AF (*n* = 4).

There were no statistically significant associations between therapy breaks and several factors, including age (≤65 vs. >65 years, *p* = 0.138), the number of previous treatment lines (TN vs. R/R, *p* = 0.244), baseline CIRS score (<6 vs. ≥6, *p* = 0.533), pre-existent AF (*p* = 0.54), hypertension (*p* = 0.709), or cardiovascular disease (*p* = 0.969) (data not shown).

Regarding the ECOG PS, a higher value (i.e., ≥2) was associated with an increased need for ibrutinib dose adjustments (odds ratio (OR): 3.4 [95% CI: 1.4–7.9]; *p* = 0.004) and a higher rate of permanent therapy discontinuation (OR: 3.5 [95% CI: 1.4–9.0]; *p* = 0.01) but not with temporary interruptions (OR: 1.5 [95% CI: 0.6–3.4]; *p* = 0.397).

The risk for permanent discontinuation due to toxicity was not increased by older age (*p* = 0.357), higher ECOG PS (*p* = 0.371), advanced Rai stage (*p* = 0.753), pre-existent cardiovascular disease (*p* = 0.756), or higher number of prior treatments (OR: 0.1 [95% CI: 0.0–0.7 CI]; *p* = 0.019), but by a CIRS score ≥ 6 (OR: 21.6 [95% CI: 2.1–226.6]; *p* = 0.011) (Table 7 and Table S2).

Death due to COVID-19 infection was reported in seven (5.7%) patients. All patients had partial or complete remission after a median (IQR) of 26 (12–41) months of treatment. Their baseline characteristics were similar to those of the study population. However, COVID-19 patients received two or more lines of therapy before ibrutinib.

A total of two del 17p patients (1.6%) had RT, leading to ibrutinib discontinuation after 8 and 41 months, and one patient’s demise during follow-up.

Among 21 (17.1%) patients who discontinued ibrutinib due to disease progression, 16 received another line of therapy, including venetoclax in 13 patients. After a median 9.5-month follow-up with salvage treatment, the estimated median PFS and OS were 18 [95% CI: 5.4–30.6] and 26 [95% CI: 2.1–49.9] months, respectively.

A total of 4 of the 11 (8.9%) patients who discontinued ibrutinib due to toxicity needed further treatment after a median of 7 (1.75–10.5) months. At 32-month median follow-up, the median PFS for patients who discontinued ibrutinib due to toxicity was 37 (15–38) months and was not statistically different from those who discontinued ibrutinib due to other reasons (*p* = 0.09, HR 2.0 [95% CI: 0.9–4.5]).

### 3.5. Adverse Events

AEs were recorded in 101 (82.1%) patients diagnosed with CLL who received ibrutinib treatment, either in the frontline or as a second or later line of therapy. A total of 71 (57.7%) and 86 (69.9%) patients displayed at least one hematological and non-hematological AE, respectively. The most common AEs were infections (29.3%), anemia (27.6%), thrombocytopenia (26%), bruising (24.4%), arterial hypertension (23.6%), neutropenia (21.1%), maculopapular rash (18.7%), and musculoskeletal pain (13.8%) (Table 8).

Compared to TN patients, R/R CLL patients had an estimated risk of developing AEs of 3.4 [95% CI: 1.1–10.7]; *p* = 0.039). A total of 38 (30.9%) patients experienced grade ≥ 3 toxicity, 5 (4.1%) of whom died due to drug-related infectious toxicity. Multivariate logistic regression analysis showed that the likelihood of grade ≥ 3 toxicity was not affected by age and CIRS score but by ECOG PS ≥ 2 (OR: 2.5 [95% CI: 1.0–6.0]; *p* = 0.044) (Table 7 and Table S2).

During follow-up, 36 (29.3%) patients developed 63 episodes of infection after a median (IQR) onset time of 9 (4.5–21) months, 11 (8.9%) of whom had a grade ≥ 3 infection (Table 7). Compared to none or one therapy line, treatment with two or more therapy lines prior to ibrutinib was associated with an increased risk of developing infections (OR: 2.97 [95% CI: 1.3–6.7]; *p* = 0.008). All patients received prophylaxis for Pneumocystis Jiroveci and the varicella-zoster virus.

In addition to infections, other non-hematological grade ≥ 3 AEs were hypertension (8.9%), bleeding (4.1%), HBV reactivation (1.6%), and AF (0.8%). The most common grade ≥ 3 cytopenia was neutropenia in 16 (13%) patients, and it was associated with fever in 6 (4.9%) cases.

Grade ≥ 3 bleeding (e.g., gastrointestinal, brain hemorrhage, hemoptysis) was reported in five (4.1%) patients, two of whom were receiving anticoagulation therapy with direct oral anticoagulant (DOAC). A total of 8 of the 11 patients treated with DOAC and ibrutinib experienced bleeding events of any grade. The risk for major bleeding was increased by concomitant anticoagulant treatment (OR 8.0 [95% CI: 1.2–54.0]; *p* = 0.013) (data not shown).

AF and atrial flutter occurred in 11 (8.9%) patients after a median (IQR) time of 12 (2–47) months. Other arrhythmias (i.e., supraventricular extrasystoles and bradycardia) were reported in 10 (8.1%) patients. AF caused treatment interruption in four patients, three of whom permanently discontinued ibrutinib. Of note, one patient had acute coronary syndrome followed by recovery and ibrutinib resuming. Drug-related AF was not associated with age ≥ 65 years (*p* = 0.228), pre-treatment hypertension (*p* = 0.868), AF (*p* = 1), or cardiovascular disease (*p* = 0.728).

In this population, there was no statistically significant association between ibrutinib-related hypertension and age, pre-existent cardiovascular comorbidities, or type-2 diabetes (data not shown).

All the patients with known pre-ibrutinib HBV infection received antiviral prophylaxis. However, HBV reactivation was reported in eight (6.5%) patients, two of whom were already receiving lamivudine prophylaxis (see also Table S1 in the Supplementary Materials for detailed prophylaxis and viral markers diagnosis). Ibrutinib was interrupted in five patients, two of whom needed permanent discontinuation.

### 3.6. Second Primary Malignancy

At the median follow-up, 16 (13%) patients were diagnosed with SPM of the skin (*n* = 4), lungs (*n* = 2), liver (*n* = 2), breast (*n* = 1), colon (*n* = 1), blood (*n* = 1), larynx (*n* = 1), and brain (*n* = 1). The median (IQR) time from ibrutinib initiation to SPM was 26 (7–37.5) months. SPM forced the discontinuation of ibrutinib therapy in six patients and eventually caused the demise of three.

### 3.7. Autoimmune Cytopenia Related to CLL

Six patients had autoimmune hemolytic anemia (AIHA), and one had pure red cell aplasia (PRCA) at the time of ibrutinib initiation. All patients responded well to ibrutinib therapy. During follow-up, neither reactivation nor new-onset cases of autoimmune phenomena were recorded.

## 4. Discussion

The present study explores the outcomes, tolerance, risk factors, and prognosis of CLL patients receiving ibrutinib outside clinical trials.

The patients were followed for a median of 37 and 19 months in the R/R CLL and TN subgroups, respectively. At the median follow-up, the median PFS for the R/R CLL was 50 months and was not reached in the TN subgroup. The clinical outcomes of this study are in line with current landmark clinical trials. However, in this study, TN patients fared slightly worse [8,25] (Table 4). Additionally, the ORR of 86.2% and the 24-month PFS (77.7% in R/R and 75.8% in TN patients) and OS rates (87.9% in R/R and NR in TN patients) were within the range of previous real-world studies (PFS 44–84.7% and OS 76.8–92.5%) [11,12,17,18,19,20].

Compared to clinical trials, the present study enrolled younger TN patients, but the overall median age of the study population was similar. In addition, our patients had fewer comorbidities (CIRS score ≥ 6 in 12 % vs. 32%) and were less pre-treated (8.2% vs. 50% received ≥3 prior therapy lines) than in clinical trials. Contrarily, 25% of our study patients had an ECOG PS ≥ 2 compared to 0–8% in RCTs [26,27]. These patient characteristics merely reflect an early selection of ibrutinib to treat CLL.

Our data attest that ibrutinib benefited patients regardless of age and prior treatments. PFS and OS were not different between TN and R/R CLL, nor between patients treated with ibrutinib in the second, third, or later line. This observation agrees with real-world data [13,20] but contrasts with RCTs data [8]. The reasons for this discrepancy may include differences in clinical and prognostic features between our cohort and the RCTs population. The del 17p, TP53 mutation, and the unmutated IGHV were detected in 37.5%, 37.5%, and 81.3% of TN patients, respectively. Indirectly, this points to ibrutinib as a first-line therapy in high-risk patients expected to have worse outcomes [28]. Nevertheless, our results may be affected by the low number of patients in the TN subgroup.

Among R/R CLL patients, we found that a TILT of fewer than 24 months was associated with poor outcomes. A short TILT reflects a poor response to chemoimmunotherapy and is a well-known predictor for reduced OS [29].

Our datasets revealed a low rate of genetic testing, particularly in the R/R setting. Therefore, the interpretation of results must be made with caution. Although our data suggested a shorter median PFS and OS in del 17p patients, this observation did not reach statistical significance. After including patients with missing FISH results, del 17p correlated with poor PFS outcomes (*p* = 0.0017). Similarly, when combining the del 17p and TP53 mutations, we observed a shorter PFS but not OS, whereas the impact of TP53 mutations alone did not reach statistical significance. The Danish population-based study obtained similar results [20]. Tedeschi A. et al. also found a statistically significant association of del 17p and/or TP53 mutations with shorter PFS and OS [30]. Conversely, several clinical trials [9,31,32], meta-analyses [33,34], and retrospective studies [12,21,35] called into question any negative impact of del 17p and TP53 mutations in ibrutinib-treated patients.

Real-world data have previously recognized the prognostic impact of ECOG PS [13,30]. In our study, a poor ECOG PS predicted shorter PFS, but OS could not be associated with any studied factors. This contrasts with the Italian experience demonstrating that a high ECOG PS strongly predicts both inferior PFS and OS [30].

We could not demonstrate a difference in PFS or OS when stratifying patients by CIRS. Nevertheless, a divergence in the PFS and OS curves was noted after 50 months of treatment, attesting that the burden of comorbidity may increase and impact outcomes with more prolonged drug exposure. Our results are at variance with Gordon et al., who found reduced event-free survival and OS in patients with a high CIRS (i.e., CIRS ≥ 7) [36].

At 32-month median follow-up, the discontinuation rate was 43.9% (54/123). Clinical trials and real-world settings such as ours have been reporting a wide spectrum of discontinuation rates with variable overlap, mostly due to differences in patient characteristics, median follow-up, treatment exposure, and AEs management.

In our study, 17.1% of patients had permanent ibrutinib discontinuation due to CLL progression, and only 8.9% had the drug stopped due to toxicity. Reasons for this favorable tolerability include the younger age, fewer comorbidities, and fewer lines of prior therapy. Our findings are comparable with RCTs data. Contrastingly, real-world data either refute [11,12,15,20,37] or confirm [14,17,18,30] a good tolerance profile.

In line with the real-life data from the UK, US, and, more recently, the Italian experience [13,15,30], we observed a significant difference in OS estimates when comparing patients who remained on treatment with those who discontinued ibrutinib. Nevertheless, discontinuation caused by toxicity and disease progression shared similar outcomes. This contrasts with other studies showing inferior OS for those with progression-related discontinuation [15].

The main toxicities leading to ibrutinib permanent cessation fit with previous reports [8,15,20,30,38]. COVID−19 infection led to the permanent cessation of ibrutinib in seven patients (5.7%), eventually causing their demise.

The incidence of RT (*n* = 2) was similar to that described in clinical trials [25,27] but in contrast with the real-world studies [12,16,18] showing higher rates.

Compared to clinical trials and real-life reports [8,9,15,17], we reported a similar drug interruption rate (38.2%) but a higher rate of dose adjustment (28.5%), possibly indicating good clinical practice. Indeed, the early discontinuation of ibrutinib was associated with an inferior outcome [13,30], whereas the temporary or permanent dose reduction did not significantly impact OS [30,39].

Age was not associated with dose reduction, dose interruption, or permanent drug discontinuation due to AEs. This observation is at variance with some real-world data [13,40] but confirms other reports that endorse ibrutinib treatment regardless of age [11,30,36]. High CIRS and ECOG PS indicated an increased risk for ibrutinib intolerance, in line with previous papers [30,36].

Comparable with published data, more than three-quarters of ibrutinib-treated CLL patients experienced at least one drug-related AE (82.1%), and one-third of patients developed grade ≥ 3 AEs (30.9%). The safety profile of ibrutinib was consistent with that previously reported by RCTs and real-world studies. Nevertheless, the AEs spectrum was different. The three most common toxicities in our cohort were infections, anemia, and bruising, whereas diarrhea, fatigue, and cough were reportedly more common elsewhere [38,41]. Notably, our study revealed a relatively high incidence (6.5%) of ibrutinib-related HBV reactivation compared to other reports (1.9%) [42], stressing the importance of HBV screening before ibrutinib initiation. We found a positive correlation between ECOG PS and grade ≥ 3 toxicity, indicating that disability and intolerance are intrinsically linked [30,36].

One-third of the patients developed 63 episodes of infection after a median time of 9 (IQR 4.5–21) months. A total of eleven (8.9%) patients had a grade ≥ 3 infection. These observations are in line with a Spanish cohort [17] but at variance with other reports [8,12,20,39], showing a higher overall incidence of infection (70%) with grade ≥ 3 infections occurring in almost half of the patients.

In this study, the AF incidence (8.9%) falls within the range of values reported by real-world studies (3–15%) [12,17] and RCTs (12–16% at 5-year follow-up) [8,38]. Similar to Dimou M. et al.’s work [19], AF was the most common toxicity leading to permanent discontinuation (*n* = 3). In contrast with other reports, preexisting cardiovascular comorbidities and diabetes were not associated with AF in our study [8]. Additionally, the frequency of new or worsened arterial hypertension (23.6%) was similar to that of RCTs (21–26%) [8,38] but higher than in other real-life CLL experiences (4.1–5.2%) [17,19].

Another specific side effect of ibrutinib is the increased bleeding risk. The on- and off-target inhibition of the BTK and TEC kinases, both involved in the platelet signaling pathways, is responsible for the excessive bleeding [43]. Our analysis reported minor (24.4%) and major (4.1%) bleeding events of incidence equal to that of landmark RCTs [8,38]. Concomitant use of anticoagulant therapy significantly increased the risk for major bleeding, consistent with other results [44].

This study had a few limitations. Firstly, it was a single-centered study. Secondly, the retrospective analysis rendered the study susceptible to missing data. Thirdly, the medical charts collected information for routine medical practice and were not formulated for research purposes. Fourthly, the number of patients treated with ibrutinib in the frontline was disproportionately small compared to the R/R CLL cohort. Finally, due to the low rate of genetic testing, the number of high-risk patients was small. Hence, the generalizability of results should be performed with caution.

## 5. Conclusions

Our findings align with the results from ibrutinib clinical trials regarding outcomes, drug breaks, and permanent discontinuation rate. The ibrutinib-related AEs were common but manageable by dose reductions and treatment breaks. The long-term survival outcomes were not impacted by age, prior treatment, or comorbidities. However, they seemed to be worse in patients with poor performance status, early relapse after pre-ibrutinib treatment, and those who permanently discontinued the drug. Patient fitness and comorbidity burden correlated with ibrutinib tolerance.

## Figures and Tables

**Table 1:** Baseline characteristics of patients at start of ibrutinib treatment in the overall population and in the treatment-naïve and R/R CLL setting.

Parameter	TN Patients (*n* = 16)	R/R CLL Patients (*n* = 107)	Overall Population (*n* = 123)
Median age (IQR), years	59 (53–68.8)	65 (59–74)	65 (58–71)
Gender, male, *n* (%)	8 (50)	68 (63.6)	76 (61.8)
ECOG PS, *n* (%) 0 1 2 3–4	5 (31.3) 11 (68.8) 0 (0) 0 (0)	14 (13.1) 62 (57.9) 28 (26.2) 3 (2.8)	19 (15.4) 73 (59.3) 28 (22.8) 3 (2.4)
Comorbidities, *n* (%) Hypertension Diabetes mellitus Cardiovascular disease ^a^ COPD Viral hepatitis B ^b^ Gastro-enteral disease Pre-existing cancer other than CLL Neurologic disease Viral hepatitis C ^b^ Biliary lithiasis Thyroid disease Permanent atrial fibrillation	5 (31.3) 1 (6.3) 2 (12.5) 2 (12.5) 2 (12.5) 0 (0.0) 2 (12.5) 0 (0.0) 0 (0) 2 (12.5) 4 (25) 0 (0.0)	48 (44.9) 25 (23.4) 37 (34.6) 11 (10.2) 10 (9.3) 7 (6.5) 5 (4.7) 4 (3.7) 3 (2.8) 3 (2.8) 2 (1.9) 3 (2.8)	53 (43.1) 26 (21.1) 39 (31.7) 13 (10.6) 12 (9.8) 7 (5.7) 7 (5.7) 4 (3.3) 3 (2.4) 5 (4.1) 6 (4.9) 3 (2.4)
Concomitant anticoagulation ^c^, *n* (%)	0 (0)	3 (2.8)	3 (2.4)
Median (IQR) CIRS score, points ≥6 points, *n* (%)	1 (0–2.75) 0 (0.0)	2 (1–4) 12 (12.1)	2 (1–4) 12 (9.8)
Rai stage, *n* (%) Stage 0-I Stage II Stage III Stage IV	1 (6.3) 7 (43.8) 5 (31.3) 3 (18.8)	9 (8.4) 51 (47.7) 17 (15.9) 30 (28)	10 (8.1) 58 (47.2) 22 (17.9) 33 (26.8)
Binet stage, *n* (%) Stage A Stage B Stage C	1 (6.3) 8 (50) 7 (43.8)	2 (1.9) 67 (62.6) 38 (35.5)	3 (2.4) 75 (61) 45 (36.6)
Number of previous therapies, *n* (%) 0 1 2 ≥3	16 (13) NA NA NA	NA 48 (44.9) 49 (45.8) 10 (9.3)	16 (13) 48 (39) 49 (39.8) 10 (8.1)
High-risk prognostic factors ^d^, *n* (%) Unmutated IGHV, missing 17p deletion, missing TP 53 mutations, missing	13 (81.3), 2 6 (37.5), 0 6 (37.5), 1	29 (27.1), 77 7 (6.5), 71 9 (8.4), 77	42 (34.1), 79 13 (10.6), 71 15 (22.2), 78
Laboratory parameters Median B_2_M (IQR), mg/LMedian LDH (IQR), UI/L Median Hemoglobin (IQR), g/dL Median Platelets (IQR), ×10^9^/L Median Creatinine (IQR), mg/dL	5.5 (3.1–6.9) 400 (260–465) 11.9 (8.7–12.5) 143 (103–176) 1.12 (0.92–1.15)	4.9 (3.8–6.2) 280 (207–371) 12.1(10.1–13.3) 125 (84–177) 0.98 (0.86–1.1)	4.9 (3.7–6.4) 296 (210–387) 12 (9.8–13.2) 126 (85–177) 0.98 (0.87–1.12)
Autoimmune CLL complications, *n* (%) AIHA PRCA	2 (12.5) 0 (0)	4 (3.7) 1 (0.9)	6 (4.9) 1 (0.8)

^a^ Including coronary heart disease, valvular heart disease, and peripheral arterial disease. ^b^ Based on serology testing (HBV antibodies and antigen and HCV antibodies). ^c^ Anticoagulation consisted of an oral new anticoagulant in all cases. ^d^ High-risk prognostic factors were not mutually exclusive. Abbreviations: TN, treatment-naïve; R/R, relapsed/refractory; IQR, 25–75th interquartile range; ECOG PS, Eastern Cooperative Oncology Group performance score; COPD, chronic obstructive pulmonary disease; CIRS, cumulative illness rating scale; CLL, chronic lymphocytic leukemia; NA, not applicable; IGHV, immunoglobulin heavy chain variable gene; B2M, beta2-microglobulin; LDH, lactate dehydrogenase; AIHA, autoimmune hemolytic anemia; PRCA, pure red cell aplasia.

**Table 2:** Prior therapy for CLL.

Regimen	First-Line Therapy *n* = 107 (100%)	Second or Later-Line Therapy *n* = 59 (55.1%)
FCR	27 (25.2)	8 (13.6)
R-CVP	22 (20.6)	15 (25.4)
R-CHOP	17 (15.9)	10 (16.9)
Clb	13 (12.1)	8 (13.6)
R-Clb	10 (9.3)	3 (5.1)
FC	8 (7.5)	6 (10.2)
G-Clb	5 (4.7)	0 (0.0)
CVP/CHOP	4 (3.7)	4 (6.8)
BR	1 (0.9)	1 (1.7)
Rituximab	1 (0.9)	0 (0.0)
Alemtuzumab	NA	1 (1.7)
Idela-R	NA	1 (1.7)
Ofatumumab	NA	1 (1.7)
Other therapies	NA	2 (3.4)

Abbreviations: CLL, chronic lymphocytic leukemia; FCR, fludarabine, cyclophosphamide, and rituximab; BR, bendamustine and rituximab; R-Clb, rituximab and chlorambucil; R-CHOP, rituximab, cyclophosphamide, doxorubicin, vincristine, and prednisone; R-CVP, rituximab, cyclophosphamide, vincristine, and prednisone; G-Clb, obinutuzumab and chlorambucil; Idela-R, idelalisib and rituximab; CVP/CHOP, cyclophosphamide, vincristine, and prednisone/ cyclophosphamide, doxorubicin, vincristine, and prednisone.

**Table 3:** Summary of the clinical response to ibrutinib in the treatment-naïve group, relapsed/refractory CLL patients, and overall population.

Type of Response	Treatment-Naïve *n* = 16	Relapsed/Refractory *n* = 107	Overall Population *n* = 123
Overall response rate, *n* (%)	14 (87.5)	92 (86)	106 (86.2)
Patient’s best response, *n* (%) Complete response ^a^ Partial response Stable disease Progression Death	8 (50) 6 (37.5) 2 (12.5) 0 (0.0) 0 (0.0)	46 (42.9) 46 (43) 14 (13.1) 1 (0.9) 27 (25.2)	54 (43.9) 52 (42.3) 16 (13) 1 (0.8) 27 (22)
Disease progression at any time after ibrutinib initiation, *n* (%)	3 (18.8)	28 (26.2)	31 (25.2)

^a^ Based on the clinical data.

**Table 4:** Comparison with pivotal clinical trials.

Study	Current Study R/R Patients	Current Study TN Patients	RESONATE Study R/R CLL	RESONATE-2 Study 1st Line
Median follow-up	37 months	19 months	72 months	96 months
Median PFS	50 months	NR	44.1 months	NR
24-month PFS rate	77.7%	75.8%	74%	89%
Median OS	NR	NR	67.7 months	NR
24-months OS rate	87.9%	NR	86%	95%

Abbreviations: R/R, relapsed/refractory; TN, treatment-naïve; PFS, progression-free survival; OS, overall survival; NR, not reached.

**Table 5:** Univariate and multivariate analysis of baseline risk factors on progression-free survival (**A**) and overall survival (**B**) in the R/R CLL patients.

A				
Variable	Univariate Model		Multivariate Model	
	Hazard Ratio	*p*-Value	Hazard Ratio	*p*-Value
Age	1.0 (1.0–1.1)	0.55	–	–
ECOG PS 2–4	2.2 (1.2–3.9)	0.01	2.7 (1.4–5.2)	0.003
Rai stage III/IV	0.9 (0.5–1.7)	0.865	–	–
Binet stage C	0.8 (0.5–1.5)	0.57	–	–
CIRS ≥ 6	1.6 (0.7–3.4)	0.251	–	–
No. of previous treatments ≥ 2	1.1 (0.6–1.9)	0.841	–	–
TILT < 24 months	6.2 (1.9–19.9)	0.002	5.7 (1.7–18.9)	0.004
IGHV unmutated *	2.0 (1.0–4)	0.042	1.4 (0.4–4.8)	0.575
Del 17p *	3.6 (1.6–8.1)	0.003	1.4 (0.5–4.3)	0.549
TP53 *	2.3 (0.9–5.8)	0.074	0.7 (0.2–2.7)	0.607
B				
Variable	Univariate Model		Multivariate Model	
	Hazard Ratio	*p*-Value	Hazard Ratio	*p*-Value
Age	1.02 (1.0–1.1)	0.20	1.0 (1–1.1)	0.436
ECOG PS 2–4	2.38 (1.1–5.1)	0.026	2.1 (0.9–4.9)	0.089
Rai stage III/IV	0.74 (0.3–1.6)	0.44	–	–
Binet stage C	0.56 (0.2–1.3)	0.170	0.5 (0.2–1.2)	0.106
CIRS ≥ 6	1.8 (0.7–4.6)	0.188	1.0 (0.4–2.7)	0.981
No. of previous treatments ≥ 2	1.2 (0.5–2.7)	0.644	–	–
TILT < 24 months	10.6 (1.4–78.1)	0.021	9.7 (1.3–70.4)	0.028
IGHV unmutated *	1.1 (0.4–3.0)	0.898	–	–
Del 17p *	1.1 (0.2–5.6)	0.924	–	–
TP53 *	0.5 (0.7–4.0)	0.536	–	–

* Patients with unknown status were also included in the analysis. Abbreviations: R/R CLL, relapsed/refractory chronic lymphocytic leukemia; ECOG PS, Eastern Cooperative Oncology Group performance score; CIRS, cumulative illness rating scale; TILT, time from initiation of the last therapy; IGHV, immunoglobulin heavy chain variable gene; Del 17p, deletion 17p.

**Table 6:** Reasons for ibrutinib cessation.

Reason for Ibrutinib Discontinuation	Frontline *n* = 16	Relapse/Refractory *n*= 107	Overall Population *n* = 123
CLL progression, *n* (%)	2 (12.5)	19 (17.8)	21 (17.1)
Other/unrelated death, *n* (%)	0 (0.0)	14 (13.1)	14 (11.4)
Toxicity, *n* (%)	2 (12.5)	9 (8.4)	11 (8.9)
Second primary malignancy, *n* (%)	0 (0.0)	6 (5.6)	6 (4.9)
Richter transformation, *n* (%)	1 (6.3)	1 (0.9)	2 (1.6%)

Abbreviations: CLL, chronic lymphocytic leukemia.

**Table 7:** Multivariate * logistic regression analysis showing the baseline factors associated with permanent discontinuation, toxicity-related permanent discontinuation, and grade ≥ 3 AEs.

Variable	Permanent Discontinuation		Toxicity-Related Permanent Discontinuation		Grade ≥ 3 AEs	
	*p*	OR (95% CI)	*p*	OR (95% CI)	*p*	OR (95% CI)
Age ≥ 65 years	0.912	1.0 (0.5–2.4)	0.357	2.1 (0.4–9.7)	0.470	1.4 (0.6–3.1)
ECOG PS ≥ 2	0.010	3.5 (1.4–9.0)	–	–	0.044	2.5 (1.0–6.0)
CIRS ≥ 6	0.025	12.0 (1.4–104.8)	0.011	21.6 (2.1–226.6)	0.503	1.6 (0.4–5.9)
No. of prior therapies ≥ 2	0.420	0.4 (0.6–3.1)	0.019	0.1 (0.0–0.7)	–	–

Abbreviations: AEs, adverse events; OR, odds ratio; CI, confidence interval; ECOG PS, Eastern Cooperative Oncology Group performance score; CIRS, cumulative illness rating scale. * For the univariate analysis, see Table S2 in the Supplementary Materials.

**Table 8:** Summary of adverse events reported for CLL patients while on ibrutinib monotherapy.

Adverse Events	Any Grade, *n* (%)	Grade ≥ 3, *n* (%)
Infections	36 (29.3)	11 (8.9)
Lung infections Sepsis Urinary tract infections Skin infections Upper respiratory infections Other infections	13 (10.6) 9 (7.3) 8 (6.5) 6 (4.9) 6 (4.9) 10 (8.0)	4 (3.3) 9 (7.3) 2 (1.6) 0 (0.0) 0 (0.0) 1 (0.8)
Non-Hematological Toxicities	86 (69.9)	22 (17.9)
Bleeding	30 (24.4)	4 (3.3)
Hypertension	29 (23.6)	11 (8.9)
Rash maculo-papular	23 (18.7)	0 (0.0)
Myalgia	17 (13.8)	0 (0.0)
Diarrhea	11 (8.9)	0 (0.0)
Atrial fibrillation/Atrial flutter	11 (8.9)	1 (0.8)
Other cardiac rhythm disorders	10 (8.1)	0 (0.0)
Hepatitis B virus reactivation	8 (6.5%)	2 (1.6%)
Arthritis/tendinitis	5 (4.1)	0 (0.0)
Gastritis	5 (4.1)	0 (0.0)
Transaminitis	5 (4.1)	1 (0.8)
Ulcerative stomatitis	4 (3.3)	1 (0.8)
Neuropathy	4 (3.3)	0 (0.0)
Hematological Toxicities	52 (42.3)	21 (17.1)
Anemia	34 (27.6)	7 (5.7)
Thrombocytopenia	32 (26.0)	3 (2.4)
Neutropenia Febrile neutropenia	26 (21.1) 6 (4.9)	16 (13) 6 (4.9)

## Data Availability

Data used in this study may be provided by the corresponding author upon reasonable request.

## Supplementary Materials

The following supporting information can be downloaded at: https://www.mdpi.com/article/10.3390/medicina59020324/s1, Figure S1: Kaplan–Meier plot showing progression-free survival and overall survival curves according to the number of treatment lines received before ibrutinib initiation (A1 and B1, respectively) and age (A2 and B2, respectively); Figure S2: Kaplan–Meier estimated progression-free survival and overall survival curves according to the TP53 mutations (A and B, respectively). Abbreviations: TP53 mut, TP53 mutations; Figure S3: Kaplan–Meier plot showing the progression-free survival and overall survival curves according to the cumulative illness rating scale (CIRS) (A and B, respectively); Figure S4: Kaplan–Meier plot showing the overall survival curves according to toxicity versus disease progression (A) and toxicity versus other reasons (B) for permanent ibrutinib discontinuation. Abbreviations: DP, disease progression; Table S1: Hepatitis B virus reactivation; Table S2: Univariate logistic regression analysis for baseline factors associated with permanent discontinuation, toxicity-related permanent discontinuation, and grade ≥ 3 AEs.
