# Supplementary material for: Real-World Clinical Outcomes and Adverse Events in Patients with Chronic Lymphocytic Leukemia Treated with Ibrutinib: A Single-Center Retrospective Study
Source: Medicina (Kaunas). 2023 Feb 9;59(2):324. doi: 10.3390/medicina59020324 (PMC9959500; doi:10.3390/medicina59020324)
Supplement: Supplementary file 1 [file medicina-59-00324-s001.zip › medicina-2145042-supplementary.pdf]

Table S1. Hepatitis B virus reactivation*

		HBV reactivation			
		No		Yes	
		HBV prophylaxis		HBV prophylaxis	
		No	Yes	No	Yes
		Count	Count	Count	Count
HBs Ag negative	Anti-HBc positive	9	2	1	0
	Anti-HBs positive	10	1	1	0
	Anti-HBc and Anti-HBs positive	7	2	1	0
	Anti-HBc and Anti-HBs negative	49	0	0	0
	Unknown	4	0	1	0
HBs Ag positive	Anti-HBc positive	1	6	1	2
	Anti-HBs positive	0	0	0	0
	Anti-HBc and Anti-HBs positive	0	1	0	0
	Anti-HBc and Anti-HBs negative	1	0	0	0
	Unknown	0	0	0	0
Unknown	Anti-HBc positive	0	0	0	0
	Anti-HBs positive	0	0	0	0
	Anti-HBc and Anti-HBs positive	0	0	0	0
	Anti-HBc and Anti-HBs negative	0	0	0	0
	Unknown	22	0	1	0

*Twelve patients were diagnosed with HBV infection prior to ibrutinib initiation. They all received viral prophylaxis (entecavir or lamivudine) during ibrutinib therapy. Two patients who were on lamivudine developed HBV reactivation. Besides, six new cases of HBV reactivation were reported during follow-up. This table summarizes the presence of diagnostic markers regarding HBV hepatitis, showing that the majority were negative for surface antigen and positive for core antibodies.

Table S2. Univariate logistic regression analysis for baseline factors associated with permanent discontinuation, toxicity-related permanent discontinuation, and grade ≥3 AEs.

Variable	Permanent discontinuation		Toxicity-related permanent discontinuation		Grade ≥3 AEs	
	p	OR (95% CI)	*p*	OR (95% CI)	*p*	OR (95% CI)
Age ≥ 65 years	0.149	1.7 (0.8-3.5)	0.080	3.4 (0.9-13.6)	0.186	1.7 (0.8-3.6)
ECOG ≥ 2	0.001	4.6 (1.8-11.1)	0.371	1.8 (0.5-6.6)	0.017	2.8 (1.2-6.6)
CIRS ≥ 6	0.007	17.4 (2.1-139.6)	0.006	7.4 (1.8-30.8)	0.141	2.4 (0.7-8.2)
No. of prior therapies ≥ 2	0.065	2.0 (1.0-4.1)	0.056	0.2 (0.0-1.0)	0.763	1.1 (0.5-2.4)
Rai stage ≥ III/IV	0.498	1.3 (0.6-2.6)	0.561	0.7 (0.2-2.5)	0.24	1.6 (0.7-3.4)

Abbreviations: AEs, adverse events; OR, odds ratio; CI, confidence interval; ECOG, Eastern Co-operative Oncology Group; CIRS, cumulative illness rating scale.

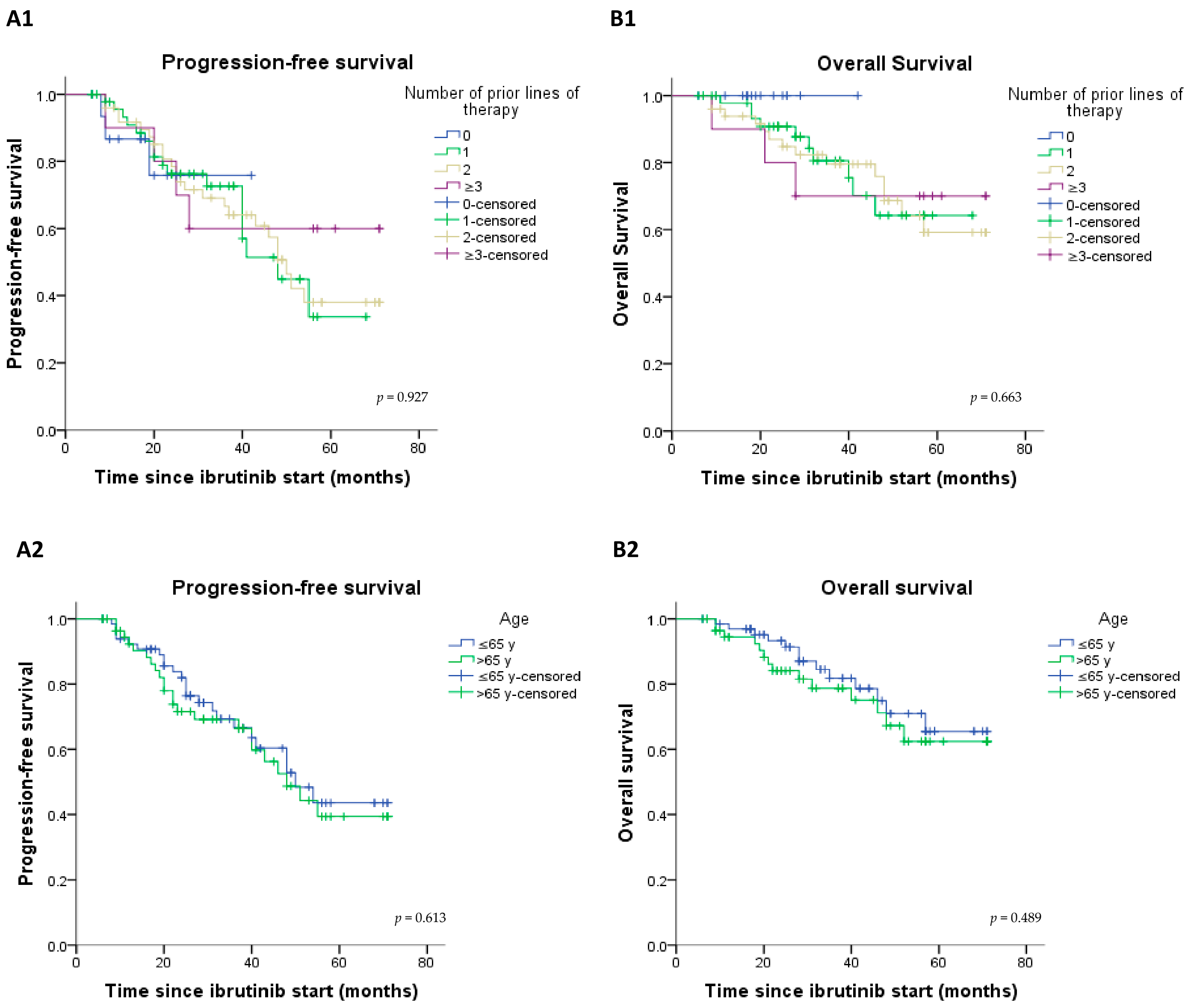

Figure S1. Kaplan–Meier plot showing progression-free survival and overall survival curves according to the number of treatment lines received before ibrutinib initiation (A1 and B1, respectively) and age (A2 and B2, respectively).

There were no differences between TN and R/R patients regarding PFS ($p = 0.348$) and OS ($p = 0.216$). The number of prior lines of therapy did not influence PFS ($p = 0.927$) nor the OS ($p = 0.663$).

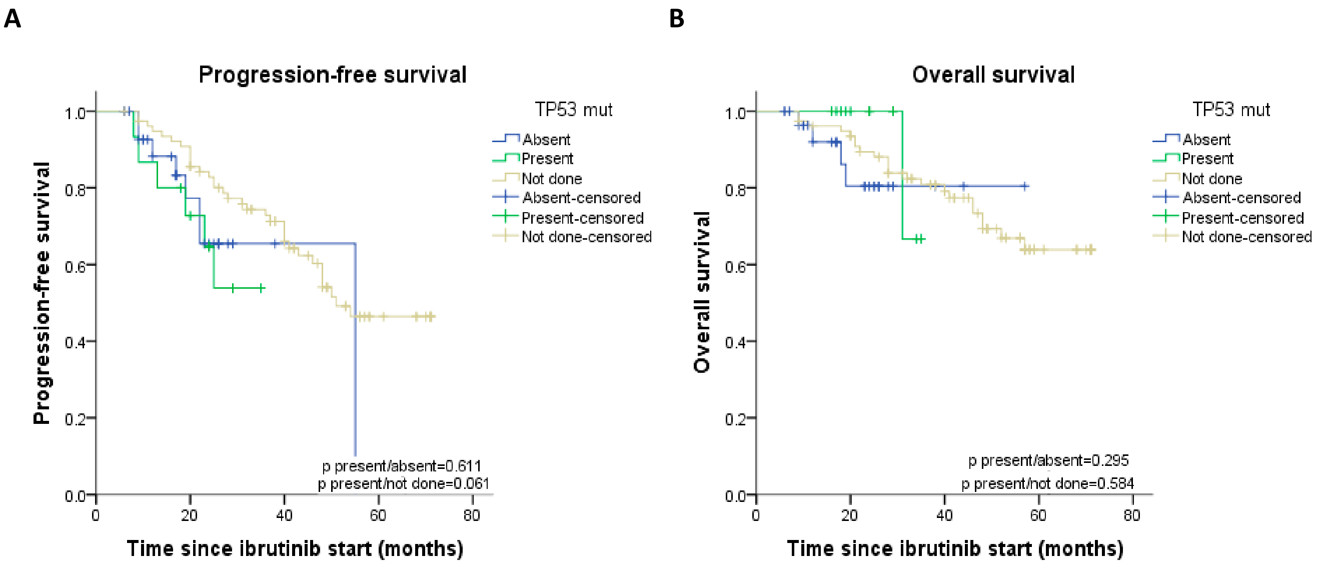

Figure S2. Kaplan–Meier estimated progression-free survival and overall survival curves according to the TP53 mutations (A and B, respectively). Abbreviations: TP53 mut, TP53 mutations.

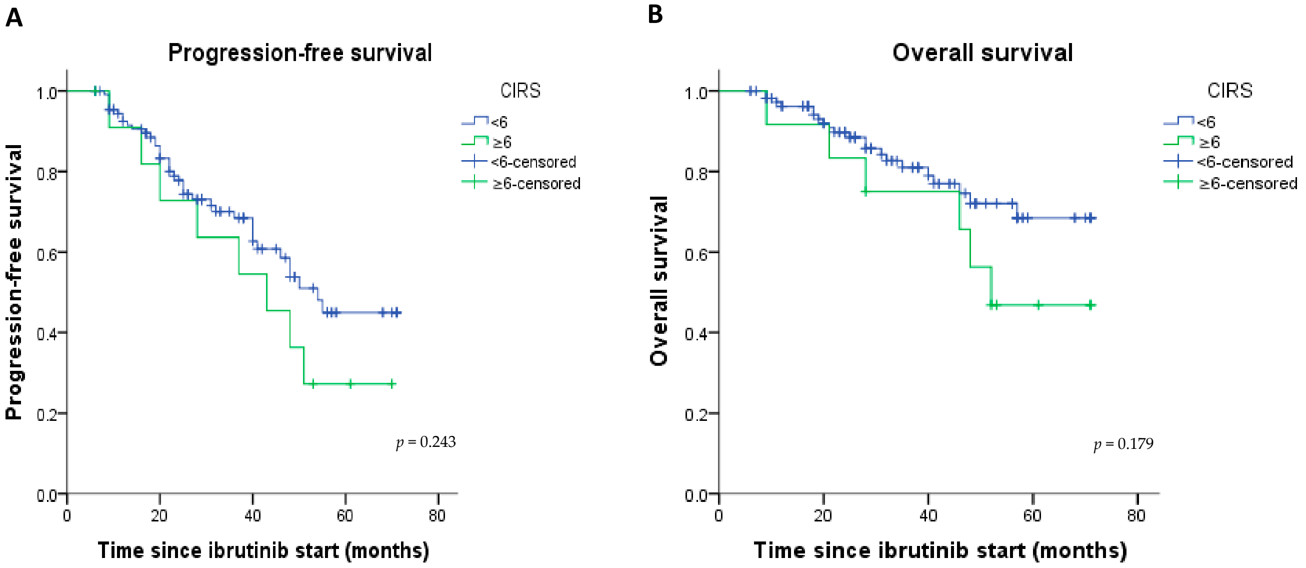

Figure S3. Kaplan–Meier plot showing the progression-free survival and overall survival curves according to the cumulative illness rating scale (CIRS) (**A** and **B**, respectively).

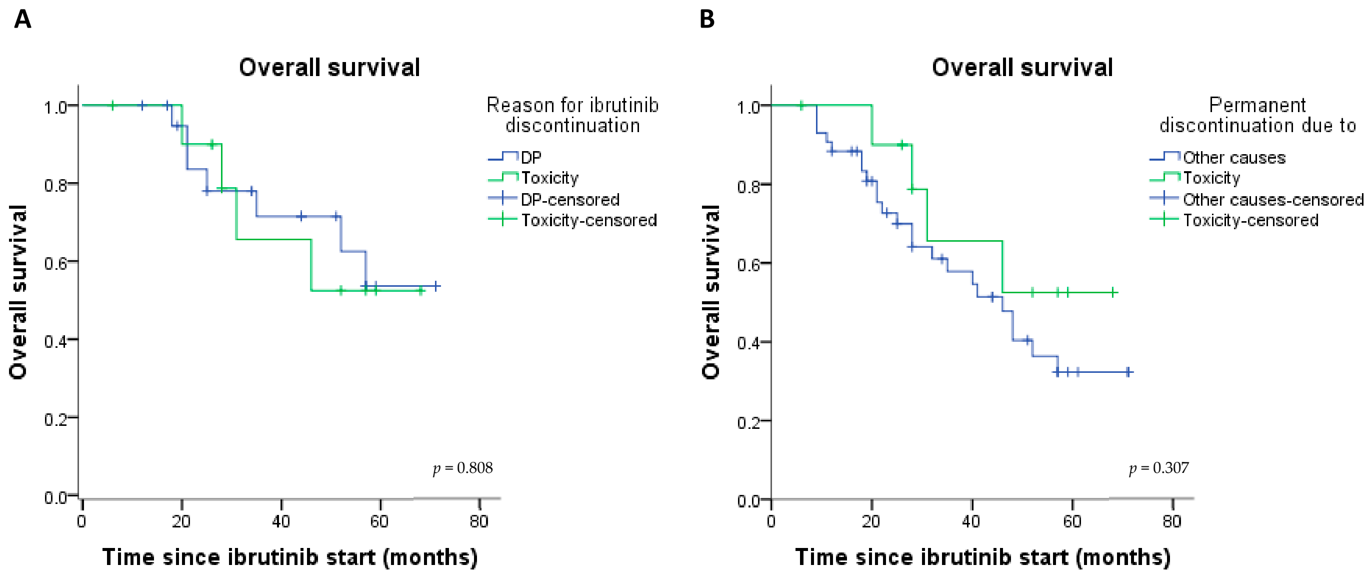

Figure S4. Kaplan–Meier plot showing the overall survival curves according to toxicity versus disease progression **(A)** and toxicity versus other reasons **(B)** for permanent ibrutinib discontinuation. Abbreviations: DP, disease progression.
